# Trans-ancestry meta-analysis of genome wide association studies of inhibitory control

**DOI:** 10.1038/s41380-023-02187-9

**Published:** 2023-07-27

**Authors:** Aurina Arnatkeviciute, Mathieu Lemire, Claire Morrison, Michael Mooney, Peter Ryabinin, Nicole M. Roslin, Molly Nikolas, James Coxon, Jeggan Tiego, Ziarih Hawi, Alex Fornito, Walter Henrik, Jean-Luc Martinot, Marie-Laure Paillère Martinot, Eric Artiges, Hugh Garavan, Joel Nigg, Naomi P. Friedman, Christie Burton, Russell Schachar, Jennifer Crosbie, Mark A. Bellgrove

**Affiliations:** 1 The Turner Institute for Brain and Mental Health, School of Psychological Sciences, Monash University, Melbourne, VIC Australia; 2 Department of Psychiatry, The Hospital for Sick Children, Toronto, ON Canada; 3 Department of Psychology and Neuroscience, University of Colorado–Boulder, Boulder, CO USA; 4 Institute for Behavioural Genetics, University of Colorado Boulder, Boulder, CO USA; 5 Department of Medical Informatics & Clinical Epidemiology, Oregon Health & Science University, Portland, OR USA; 6 Knight Cancer Institute, Oregon Health & Science University, Portland, OR USA; 7 Department of Psychological and Brain Sciences, University of Iowa, Iowa City, IA 52242 USA; 8 Department of Psychiatry and Psychotherapy, Charité-Universitätsmedizin Berlin, corporate member of Freie Universität Berlin and Humboldt-Universität zu, Berlin, Germany; 9 Institut National de la Santé et de la Recherche Médicale, INSERM U1299 “Developmental trajectories & psychiatry” Université Paris-Saclay, Ecole Normale supérieure Paris-Saclay, CNRS, Centre Borelli, Gif-sur-Yvette, France; 10 AP-HP, Sorbonne Université, Department of Child and Adolescent Psychiatry, Pitié-Salpêtrière Hospital, Paris, France; 11 Etablissement Public de Santé (EPS) Barthélemy Durand, 91700 Sainte-Geneviève-des-Bois, France; 12 Departments of Psychiatry and Psychology, University of Vermont, 05405 Burlington, VT USA; 13 Division of Psychology, Department of Psychiatry, Oregon Health & Science University, Portland, OR USA

**Keywords:** Neuroscience, Psychology

## Abstract

Deficits in effective executive function, including inhibitory control are associated with risk for a number of psychiatric disorders and significantly impact everyday functioning. These complex traits have been proposed to serve as endophenotypes, however, their genetic architecture is not yet well understood. To identify the common genetic variation associated with inhibitory control in the general population we performed the first trans-ancestry genome wide association study (GWAS) combining data across 8 sites and four ancestries (*N* = 14,877) using cognitive traits derived from the stop-signal task, namely – go reaction time (GoRT), go reaction time variability (GoRT SD) and stop signal reaction time (SSRT). Although we did not identify genome wide significant associations for any of the three traits, GoRT SD and SSRT demonstrated significant and similar SNP heritability of 8.2%, indicative of an influence of genetic factors. Power analyses demonstrated that the number of common causal variants contributing to the heritability of these phenotypes is relatively high and larger sample sizes are necessary to robustly identify associations. In Europeans, the polygenic risk for ADHD was significantly associated with GoRT SD and the polygenic risk for schizophrenia was associated with GoRT, while in East Asians polygenic risk for schizophrenia was associated with SSRT. These results support the potential of executive function measures as endophenotypes of neuropsychiatric disorders. Together these findings provide the first evidence indicating the influence of common genetic variation in the genetic architecture of inhibitory control quantified using objective behavioural traits derived from the stop-signal task.

## Introduction

Executive functions (EF) are essential in our everyday lives and critical for goal-directed behaviour. We need to adjust our actions based on changes in the environment, direct attention towards particular tasks, monitor performance and inhibit irrelevant or automatic impulses. Broadly, these executive functions can be conceptualised as falling into three main categories – cognitive flexibility, working memory, and inhibitory control [1]. Whereas EFs are linked to a range of positive outcomes such as educational attainment [2], quality of life [3, 4], fewer behavioural problems [5], and general health-related behaviours [6], impairments in these cognitive processes are associated with risk for several psychiatric and neurodevelopmental disorders (NDDs) including attention deficit hyperactivity disorder (ADHD) [7–9], autism spectrum disorder (ASD) [10], obsessive-compulsive disorder (OCD) [11–13], and schizophrenia [14, 15].

Inhibitory control presents a particular facet of executive functioning that is directed at inhibiting inappropriate or irrelevant responses involving a set of distinct cognitive processes such as the ability to selectively control attention and behaviour as well as override the innate predisposition for a prompted action. Inhibitory control can be assessed in a laboratory setting using the stop signal paradigm [16, 17], in which participants typically perform a “go” task but in a minority of the trials are presented with a stop signal that requires them to withhold an already initiated response to a go-signal. The performance in a stop-signal task is therefore modelled as a race between the initiated ‘go process’ that is triggered by a frequently presented go-stimulus and a ‘stop process’ which is triggered by the stop-signal, such that the response is inhibited if the stop process finishes before the go process [18]. As a result, the performance on the stop signal task is characterised by three main measures: mean go reaction time (Go RT) reflecting the overall processing speed for go-stimuli, go reaction time variability (Go RT SD) corresponding to the efficiency with which top-down regulation of attention can be exerted over behaviour [19], and the stop signal reaction time (SSRT) which quantifies the efficiency of response inhibition, with longer SSRTs indicative of poorer response inhibition [16].

Deficits in inhibitory control and associated cognitive measures are common features in heritable neurodevelopmental disorders (NDDs) such as ADHD, ASD and schizophrenia [9, 20–22]. Executive functions in general, and measures of inhibitory control in particular, serve as main candidate endophenotypes for ADHD [23–25] and have been proposed for ASD and schizophrenia [26, 27]. Convergent evidence to date suggests that inhibitory control is also under the genetic influence with moderate heritability estimates ranging from h^2^ = 0.2–0.6 identified across a range of inhibitory control measures, including the stroop task [28, 29], stop signal task [28, 30, 31], go/no-go task [32], prohibition task [33], and the antisaccade task [28, 34]. Moreover, a latent variable derived from a combination of inhibitory control measures was almost entirely genetic in origin [28].

Supplementing these behavioural findings, inhibition-related event components derived from electroencephalography (EEG) also demonstrate heritability of 0.5–0.6, further supporting the role of genetic influences in inhibitory control [35]. Bivariate heritability analyses indicate shared genetic influences between ADHD traits and the primary index of the efficiency of response inhibition derived from the stop-signal task, SSRT, suggesting the potential for common genetic contributions to these two phenotypes [30]. There is some evidence of co-heritability of executive function and inhibitory processing measures with schizophrenia [36, 37], as well as familial presentations in ASD [38]. Further research is needed to examine the genetic sharing between NDDs and inhibitory control and its potential as an endophenotype.

The demonstrated role of genetics in inhibitory control supports further investigations into the specific genes associated with this executive function that could help to determine contributing neurobiological mechanisms for these processes and associated disorders. Determining such genes so far has been a challenge with suggestive associations identified mainly through candidate gene studies linking response inhibition to genetic variants in a number of genes such as the adrenergic receptor genes *ADRA2A* [39] and *ADRA2B* [40], norepinephrine transporter gene *SLC6A2* [41, 42], dopamine transporter gene *DAT1* [43, 44], dopamine receptor gene *DRD2* [45], serotonin type 2 A receptor gene *HTR2A* [46], and neuronal tryptophan hydroxylase-2 gene *TPH2* [47]. Candidate gene studies, however, have been extensively criticised due to high false-positive rates [48] and poor reproducibility [48, 49]. Indeed, a later study failed to identify any conclusive associations for any of the seven a priori single nucleotide polymorphisms (SNPs) previously associated with stop signal task performance [50]. Therefore, more systematic and agnostic approaches may be required to establish robust associations.

In contrast to candidate gene studies where genetic variants are selected a priori, genome-wide association studies (GWAS) provide a systematic approach to identifying genetic associations in a data-driven way, as well as allowing quantification of the extent of genetic influences attributable to common genetic variation. Several GWASs to date have investigated different aspects of executive functioning including processing speed [51–53], and the latent measures of working memory and inhibitory control [52], however very few genome-wide significant associations have been identified. The largest and most recent GWAS of executive function investigated the common executive function factor score (cEF) derived from multiple tasks in the UK Biobank dataset and found 129 independent lead variants mainly associated with fast synaptic transmission [54]. SNP-heritability studies indicate that common genetic variation explains a substantial fraction of variance in working memory ($h_{SNP}^2$ = 0.3) [52] and processing speed ($h_{SNP}^2$ = 0.11–0.19) [51, 52] suggesting that with enough power one can expect to identify more genome-wide significant associations that could inform the genetic mechanisms of different executive functions, including inhibitory control.

Here we performed the first trans-ancestry GWAS meta-analysis of inhibitory control in a general population sample of up to 14,877 participants, focusing on executive control measures derived from the stop-signal task. Go trial reaction time (GoRT) quantified processing speed, go reaction time variability (GoRT SD) quantified the efficiency of top-down regulation of attention, and stop signal reaction time (SSRT) served as a measure of response inhibition. Given the stark lack of diversity in GWAS, the inclusion of participants beyond solely those of European descent is needed to ensure representativeness, even as sample sizes are still growing [55]. Although we did not identify significant genome-wide hits for any of these phenotypes, the significant SNP heritability estimates for both response variability and response inhibition indicate that interindividual differences in both of these measures are influenced by genetic factors. Power analyses showed that in this study we had excellent power to detect at least one association at genome-wide significance if the number of common causal variants was ≤500. Our failure to identify genome-wide associations suggests that the actual number of contributing variants is significantly greater and larger sample sizes are necessary to identify robust associations. We also showed that in Europeans the polygenic risk for ADHD was significantly associated with reaction time variability, and the polygenic risk for schizophrenia was significantly associated with go reaction time, while in East Asians polygenic risk for schizophrenia was associated with response inhibition, further supporting the suggested utility of executive functions as endophenotypes.

## Methods

### Participants

In this study we aggregated data across eight independent samples from the general population [Spit1, Spit2, Adolescent Brain Cognitive Development℠ Study (ABCD Study^**®**^), MELBOURNE, IMAGEN, COLORADO, Michigan-ADHD-1000, Oregon-ADHD-1000] and four ancestral groups [African (AFR), East Asian (EAS), European (EUR), South Asian (SAS)], totalling to 14,877 participants. Spit For Science (Spit1, Spit2) is an ongoing study at The Hospital for Sick Children in Toronto (Canada) aiming to investigate the genetics of cognition, physical health and well-being in children aged 6–17 years [30, 56]. The ABCD Study is a publicly available longitudinal dataset from the USA containing participants aged 9 to 10 years at their baseline assessment, focusing on cognition, brain development, and mental and physical health [57, 58]. The Melbourne sample (MELBOURNE) is derived from an ongoing study at Monash University in Melbourne, Australia that is designed to systematically assess neurocognition, psychopathological symptoms, genetics, as well as brain structure and function in a large sample of healthy young adults aged 18–50 years [59]. The IMAGEN sample was derived from the longitudinal IMAGEN dataset collected across eight centres in Europe combining brain imaging, genetics and psychiatry to understand brain development and behaviour in adolescents aged 14 years at baseline [42]. The Colorado sample (COLORADO) includes same sex monozygotic (MZ) and dizygotic (DZ) twins recruited from the Colorado Longitudinal Twin Sample that was designed to investigate genetic and environmental influences on cognitive and emotional development [60, 61]. The Oregon-ADHD-1000 (OREGON) is a community-recruited, longitudinal, case-control cohort of children (age 7–11 years at baseline) from northwest Oregon (USA) that is enriched for psychopathology [62–66]. The Michigan-ADHD-1000 (MICHIGAN) is a cohort of youth (age 6–21 years) with the same recruitment and assessment procedures as the OREGON cohort, but recruited from a different demographic population (central Michigan, USA) [67, 68]. Only control subjects were selected for analysis from both of the latter cohorts.

### Phenotypes

To investigate the genetics of executive function we selected three behavioural traits derived from the stop-signal task (SST) [69], namely, mean go reaction time (GoRT), go reaction time variability (GoRT SD) and stop signal reaction time (SSRT) representing overall processing speed, response variability, and response inhibition, respectively. All stop signal tasks consisted of two types of trials: “go” trials and “stop” trials. In a “go” trial participants are asked to respond to a stimulus as quickly and as accurately as possible by a button press corresponding to a particular stimulus. In “stop” trials participants are required to suppress their response to a go stimulus after the stop stimulus is presented therefore inhibiting an already initiated process. Stop signal tasks were administered independently between studies according to the site-specific study design and best practices (for the experimental procedures in each study, see Supplementary Text S1; for the description of the SSRT integration method, see Supplementary Text S2).

### Genotyping and imputation

Samples were genotyped on a variety of arrays that are listed in Supplementary Table S1. For Spit 1&2 studies, only participants for which all 4 grandparents shared the same ancestry (either EUR, EAS or SAS) were genotyped. For the ABCD Study, we restricted analyses to non-Hispanic EUR, EAS, SAS and AFR ancestries. Recruitment for all other study cohorts was restricted to participants of EUR ancestry. Genotyping quality control (QC) was performed by different study centres according to their own best practice and pipelines (for genotyping and QC details for each site see Supplementary Text S3).

Imputation was performed separately for all studies and genotyping arrays, using data from phase 3, version 5 of the 1000 Genomes project for reference. Data for Spit 1&2 and ABCD Study[Go] were imputed using Beagle v4.1 [70]. Data for MELBOURNE, IMAGEN and ABCD Study[SSRT] were imputed using minimac v4 on the Michigan imputation server [71]. The COLORADO sample was imputed on the Michigan Imputation Server using minimac v4, Eagle v2.4 for phasing. Dosage data were used for all these sites. For both OREGON and MICHIGAN, non-genotyped SNPs were imputed with the same procedure using IMPUTE2 [72]; autosomal chromosomes were pre-processed and phased using SHAPEIT [73]. Variant positions and alleles were checked against the reference panel and SNPs that were missing or mismatches were removed. Genotype probabilities for these two sites were converted to best-guess genotypes with the genotype set to missing if the probability was <0.8.

### Association analysis

Association analyses were performed within each study and within each ancestral group, focusing on SNVs with MAF > 1% and imputation quality r^2^ > 0.80. Most studies used allele dosage, while data in OREGON and MICHIGAN samples were based on the best-guess genotype calls (i.e. from reading vcf files into plink). To account for relatedness between participants, we used linear mixed models implemented in GEMMA v0.98.1 [74]. All traits (mean GoRT, GoRT SD, SSRT) were analysed on the natural log scale. We used sex, age, age^2^ and age x sex as covariates, as well as the first 3 principal components constructed from the SNP data. An example from the Spit1 study demonstrates that 3 principal components were sufficient to cluster regional ancestries within continental ancestries (see Supplementary Fig. S1).

Within ancestral groups, the studies were meta-analysed using METAL release 2011-03-25 [75], with a focus on SNVs covering >70% of the samples, as was done elsewhere [76]. Summary statistics from each site and ancestral group were meta-analysed using the methods described in [77] and originally implemented in MR-MEGA v0.1.5. Briefly, the method accounts for the possible heterogeneity of the effect sizes of an SNV in different ancestries by modelling in a regression framework the individual study effect sizes as a function of axes of genetic variation computed from multidimensional scaling. We used 3 axes of variation in addition to the regression intercept to model our 4 ancestral groups. For each SNP in study *s*, the observed effect size (*β*_*s*_) was estimated as:

$$\beta _s = a + b_1x_{1s} + b_2x_{2s} + b_3x_{3s} + \epsilon _s$$

where *x*_1*s*_, *x*_2*s*_ and *x*_3*s*_ are the (pre-computed) values of study *s* in the 3 axes of variation (Supplementary Fig. S2). Each study is weighted according to the inverse of the variance of its effect size. Significance is obtained from testing *a* = *b*_1_ = *b*_2_ = *b*_3_ = 0, in which case the observed effect sizes in each study are no different from random residuals (*ϵ*_*s*_). The original implementation of MR-MEGA can only analyse complete data, so we implemented our own regression in R to allow for missing results in some of the studies that arose due to frequency or imputation quality thresholds. We verified that results from our code and MR-MEGA agree for complete data. Axes of genetic variation were calculated using MR-MEGA from SNPs with complete data.

Gene-based analysis was performed using MAGMA version 1.10 [78], using the auxiliary files available on the software’s website (19,427 genes). Since the analysis depends on LD patterns, it was performed separately within each ancestry group, then the results were combined using Stouffer’s method. Pathway analysis was also performed with MAGMA, using curated gene sets (collection C2) downloaded from Molecular Signatures Database (MSigDB), v2023.1.Hs [79] (https://www.gsea-msigdb.org/gsea/msigdb/), restricting to sets with 10 to 1000 genes (5637 sets). A Bonferroni correction was used to account for multiple testing for the gene-based and pathway-based analyses.

### SNP heritability, genetic correlations and polygenic scores

We assessed SNP heritability ($h_{SNP}^2$) for each phenotype (mean GoRT, GoRT SD and SSRT) as well as the genetic correlation between each of those phenotypes using LD score regression as implemented in LDSC v1.0.0 [80]. We restricted these analyses to SNVs with complete data to ensure that results were not affected by imbalances in power between studies. We used the LD scores as provided within LDSC v1.0.0 for EUR and EAS samples and performed our own calculations of scores for AFR and SAS samples using the same methods. We investigated trans-ancestry genetic correlations using POPCORN (installed from git commit #facdfbc) [81].

Polygenic scores (PGS) were constructed from summary statistics derived from GWAS for ADHD [76], ASD [82], and SCZ [83] using a pruning and thresholding approach as implemented in PLINK [84] and PRSice v1.25 [85], clumping SNPs for LD (using default *r*^*2*^ < 0.1 in 250 kb windows). Analysis of the European ancestry cohorts excluded the data from COLORADO, a twin study. Summary statistics were also available in EAS samples for SCZ, which were used with the EAS ancestry samples from Spit1, Spit2 and ABCD Study. PGS were tested for association in our samples with our traits, evaluated at the p-value thresholds 0.001, 0.05, 0.10, 0.20, 0.30, 0.40, 0.50. We restricted these analyses to SNVs with imputation quality r^2^ > 0.8. PGS effect sizes between studies were meta-analysed using fixed-effect, inverse variance methods. To account for testing multiple correlated PGS derived from the p-value inclusion thresholds, we calculated an effective number of independent PGS from the data and applied a Bonferroni correction with respect to that number (for a description, see Supplementary Text S5). We chose this approach of correcting for multiple testing because constraints on sharing individual level data precluded the use of permutation procedures. Multiple testing thresholds were calculated separately in EUR and EAS analyses. Although we would be interested in testing the association of our cognitive traits with a PGS based on OCD, the largest publicly available GWAS [86] is too small to provide good estimates.

## Results

The total sample for each respective GWAS consisted of 14 844 subjects for GoRT SD, 14 877 for mean GoRT and 14 114 for SSRT (descriptive characteristics for each study are shown in Table 1). Samples from the different study centres and ancestries were generally comparable in terms of age and sex, with a few exceptions. ABCD Study had slightly younger participants with an age range that was narrower compared to other studies, whereas MELBOURNE and COLORADO studies consisted of young adult participants.

**Table 1:** Descriptive statistics for each ancestry and study sample.

Ancestry	Study	*N* GoRT SD	*N* Mean GoRT	*N* SSRT	Mean age (SD)	Females %
AFR	ABCD Study	781	781	706	9.95 (0.61)	52
EAS	ABCD Study	97	97	89	9.91 (0.63)	55
	Spit1	847	847	847	11.51 (3.00)	52
	Spit2	294	294	294	10.26 (3.16)	55
	TOTAL	1238	1238	1230		
EUR	ABCD Study	3844	3844	3577	9.95 (0.62)	48
	Spit1	4943	4943	4943	11.03 (2.75)	48
	Spit2	727	727	727	10.29 (3.03)	49
	MELBOURNE	942	942	668	22.42 (4.89)	57
	IMAGEN	1123	1123	1074	13.7 (3.39)	48
	COLORADO	524	524	524	22.59 (1.11)	53
	OREGEON	159	159	155	9.43 (1.52)	44
	MICHIGAN	97	130	47	13.05 (3.21)	48
	TOTAL	12359	12392	11715		
SAS	ABCD Study	32	32	29	10.05 (0.72)	38
	Spit1	250	250	250	11.52 (3.07)	50
	Spit2	184	184	184	10.51 (3.39)	49
	TOTAL	466	466	463		
	GRAND TOTAL	14844	14877	14114		

Sample sizes for each of the analysed phenotypes (GoRT SD, mean GoRT, and SSRT). Also shown are statistics for the covariates age and sex, the latter being expressed as a percentage of females. *AFR – * African ancestry, *EAS* – East Asian ancestry, *EUR* – European ancestry, *SAS* – South Asian ancestry.

### Association analyses

**Fig. 1:**
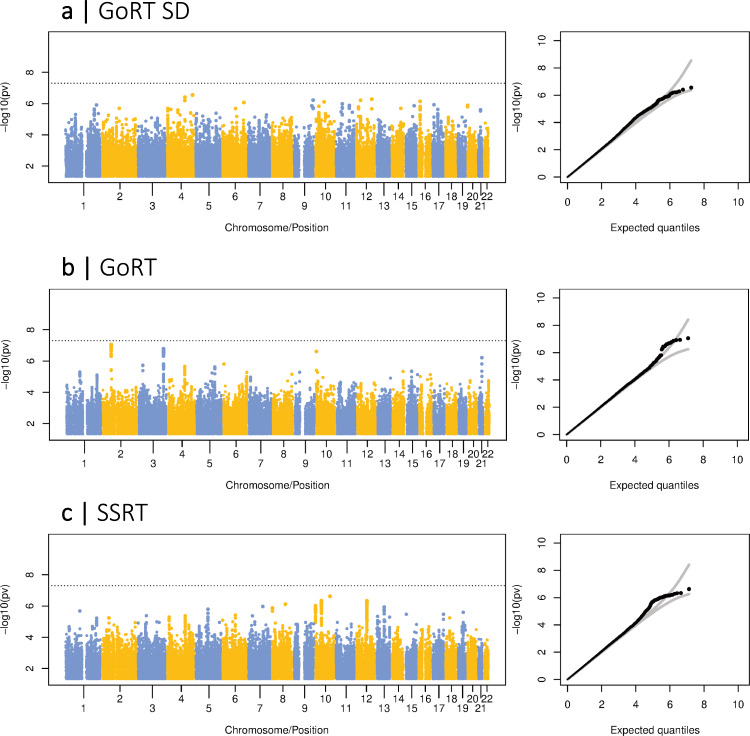
Trans-ancestry GWAS. Manhattan plots and corresponding quantile-quantile (QQ) plots for GoRT SD (**a**); mean GoRT (**b**); SSRT (**c**). Dashed lines on the Manhattan plots indicate *p* < 5 × 10^–8^ threshold. Grey lines on the QQ plots represent 95% confidence bands.

First, we performed trans-ancestry GWASs for each phenotype and found that no variant reached genome-wide significance (*p* < 5 × 10^–8^) for any of the studied traits (Fig. 1, Supplementary Figs. S3–5 represent ancestry-specific analyses). A total of 17 regions had lead SNPs meeting suggestive significance (*p* < 10^–6^): 8 for GoRT SD, 4 for GoRT mean, and 6 for SSRT (see Supplementary Table S2). Regional plots are shown in Supplementary Fig. S6a–h. Based on the investigation of LD score regression intercepts in the largest sample (EUR) we found that the potential biases caused by insufficiently controlled fine-scaled ancestry or cryptic relatedness were not significant for either GoRT SD or SSRT, indicating that association tests were not inflated (or deflated) (Table 2). Considering this result, the significant intercept deviation from 1 observed in the case of mean GoRT can be treated as spurious. Another possibility is that the genetic architecture of mean GoRT comprises predominantly rare causal variants, which are known to produce higher intercepts and negative slopes [80]. In some cases, other ancestries also demonstrated intercepts exceeding 1 (depending on the trait), likely owing to admixture in these populations or small sample sizes (Supplementary Table S3). Similar results were obtained using 10 PCs as covariates (not shown). Gene-based and pathway-based analyses also did not identify any genome-wide significant results, using a Bonferroni correction based on the number of analysed genes or gene sets, for any of the three traits (see Supplementary Fig. S7 and Supplementary Text S4).

**Table 2:** Heritability estimates for each phenotype in European ancestry subjects.

Trait	h2 (constrained int.)			h2 (unconstrained int.)			Intercept		
	Estimate	SE	*P*-value	Estimate	SE	*P*-value	Estimate	SE	*P*-value
GoRT SD	**0.082**	**0.029**	**0.002**	0.065	0.050	0.096	1.005	0.012	0.675
GoRT Mean	0.040	0.028	0.072	−0.089	0.046	0.974	1.038	0.012	0.001
SSRT	**0.081**	**0.031**	**0.004**	0.008	0.053	0.440	1.020	0.013	0.125

LD score regression estimates, standard error (SE) and significance (*P*-value) of the proportion of trait variance (*h*^2^) and regression intercept explained by common variants. When the regression intercept is constrained, it is constrained to 1. Tests are one-sided for *h*^2^ ( > 0) and two-sided for the intercept ( ≠ 1). Heritability estimates presented in bold are considered statistically significant.

### Heritability and association with polygenic scores

Next, we evaluated the combined effect of common genetic variation for each phenotype by calculating SNP heritability ($h_{SNP}^2$) focusing on the largest available sample (EUR) (Table 2). Both GoRT SD and SSRT showed significant and similar SNP heritabilities of ~8.2% (*p* = 0.002 and *p* = 0.004, respectively, when the intercept was constrained to reduce the variability). LD score regression intercept significantly departing from 1 would indicate a non-negligible impact of confounding factors such as cryptic relatedness and population stratification [80]. In both cases, the intercept was not significantly different from 1, motivating the constraint. When the LD score intercept was free to vary, the point estimate for the GoRT SD was reasonably robust, albeit not significant ($h_{SNP}^2$ = 0.065, *p* = 0.096), whereas, for SSRT the effect of the constraint was critical ($h_{SNP}^2$ = 0.008 with unconstrained intercept, *p* = 0.44). For completeness, Supplemental Table S3 shows heritability for other ancestries but owing to the relatively small sample sizes these estimates should be considered with this limitation in mind. We also investigated trans-ancestry genetic correlations between phenotypes in the two largest ancestral groups (EUR and EAS), however, due to small the sample sizes, the standard error of the trans-ancestry genetic correlation estimate (a parameter bounded by 1) was above 10, making inference and interpretation uninformative.

**Fig. 2:**
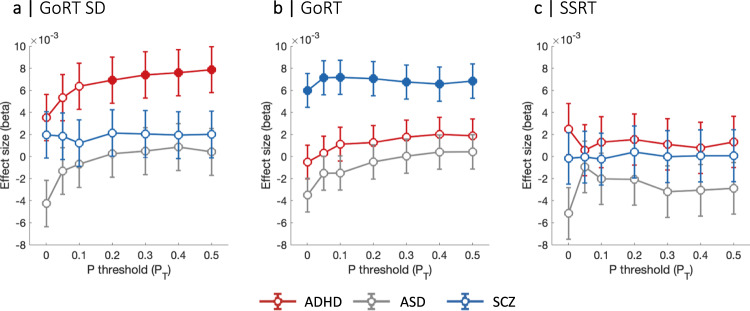
ADHD, Autism Spectrum Disorder and Schizophrenia polygenic risk score associations in European Ancestry. Associations between PRS for ADHD, ASD, and schizophrenia and GoRT SD (**a**), GoRT (**b**), and SSRT (**c**) based on the meta-analysis of EUR samples. Each subplot represents the estimated effect sizes (beta) and standard error (se) across a range of p-value thresholds (P_T_). Filled circles indicate association *p*-values that pass Bonferroni correction for multiple testing for the 3 traits (*p* < 0.0019).

To evaluate the relationships between executive function and the genetic risk for each of ADHD, ASD, and SCZ we constructed polygenic scores based on appropriate PGC summary statistics using samples of EUR ancestry as well as EAS whenever possible. The associations between the PRS and each of the behavioural measures were performed in each study centre separately and the effect sizes of the PGS (standardised to have unit variance) on the traits were meta-analysed. We found that, in EUR, ADHD PGS were significantly associated with GoRT SD, but did not show any associations with GoRT or SSRT (Fig. 2, see Supplementary Table S4 for more detailed results). The largest and most significant effect of the PGS on GoRT SD ($\hat \beta$ = 0.0079, se=0.0021; *p* = 0.000123) was observed using a clumped set of SNPs retaining variants with *p* < 0.5 based on the PGC ADHD GWAS, where larger PGS (representing the increased risk of ADHD) were associated with larger variability of the Go trial responses. This result was mostly driven by the ABCD Study cohort (*p* = 0.000126) and showed considerable (*p* = 0.051) heterogeneity between studies (forest plot shown in Supplemental Fig. S8). We also found that in EUR, SCZ PGS were significantly associated with GoRT mean across all p-value thresholds ($\hat \beta$ = 0.0071, se = 0.0015; *p* = 3.39 × 10^−6^), but not GoRT SD (p_min_ = 0.31) or SSRT (p_min_ = 0.86). Increased genetic risk of SCZ was consistently associated with larger GoRT scores across studies (minimum *p*-value for heterogeneity = 0.065, forest plot shown in Supplemental Fig. S9). We calculated that the correlated PGS for the seven tested *p*-value thresholds per GWAS corresponded to an effective number of independent variables equal to ~3 (*N*_*e*_ = 3.00 in ABCD Study; *N*_*e*_ = 2.96 in Spit1, both for ADHD), which leads to a Bonferroni corrected threshold of 0.05/(3 tests × 3 traits × 3 GWAS studies) = 0.0019. This means that the observed associations between ADHD PGS and GoRT SD (*p* = 0.00012) and SCZ PRS and GoRT mean (*p* = 3.39 × 10^−6^) are both significant after multiple testing corrections. In the EAS-specific analysis, the PGS for SCZ was significantly associated with SSRT (*p* = 0.0046), compared to a Bonferroni threshold of 0.05/3 tests × 3 traits = 0.0056, with a positive direction of effect (Supplementary Fig. S10, forest plot shown in Supplemental Fig. S11).

### Power analyses

In order to assess the power for detecting at least one association with a common (MAF > 1% in EUR as baseline) causal variant (CV – defined here as the variant that is responsible for the association signal at a particular locus) at genome-wide significance, we performed a simulation study. Leveraging the significant and robust heritability for GoRT SD, we aimed to simulate a varying number of CVs, together explaining 8.2% of the variance of a simulated, normal trait. CVs were randomly selected among those with MAF > 1% in the EUR population of the 1000 Genomes project and were assigned effect sizes drawn from a normal distribution and neutral selection. From a larger set of pre-simulated whole genomes, we randomly selected genotype data for 12359 EUR, 1238 EAS, 466 SAS and 781 AFR samples, constructed the polygenic score from the causal ones and generated a trait by adding an environmental variance appropriately scaled (see Supplementary Text S6). For the effect sizes, we simulated two scenarios: one where the effect sizes are the same in all ancestries, and one where the effect sizes are uncorrelated between ancestries. CVs were taken to be the same, for parsimony. Details of the simulation designs are provided in Supplementary Text S6.

**Fig. 3:**
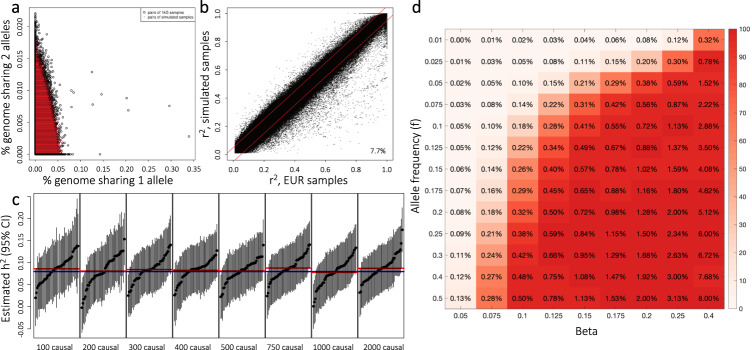
Validation of simulated replicates and the power to detect association for a single SNV. **a** Percentage of genome shared identical by descent between pairs of 503 EUR samples from the 1000 Genomes project (1 kG; black circles) and between pairs of 10000 simulated samples derived from them (red dots). **b** Linkage disequilibrium (r^2^) between pairs of SNPs calculated in 503 EUR samples from 1 kG (*x*-axis) compared to (size-matched) 503 simulated samples (*y*-axis). Red bands indicate differences of +/− 0.05; 7.7% of SNP pairs fall outside the bands. **c** Estimated LDSC heritability calculated from 12,359 simulated samples of EUR ancestry, for a trait simulated to have 8% heritability (blue horizontal line). A number of simulated causal variants are indicated on the horizontal axis. Red lines represent mean estimates, calculated from 100 simulated replicates. Vertical lines represent 95% confidence intervals for the heritability estimates (black points). **d** The power to detect association for a single SNV. The colours in the matrix represent the power (R2) to detect an association at genome-wide significance between a SNV and a unit-variance trait for varying allele frequency and effect size (beta: increase in trait value per minor allele). Values in each cell correspond to the percentage of trait variance explained by that SNV. R2 is calculated to be 2*Beta^2*f*(1-f).

We show that the simulated whole genomes are: (i) indistinguishable from unrelated samples (Fig. 3a), (ii) that they closely preserve the LD structure of the original 1000 Genomes samples they are derived from (Fig. 3b), and that (iii) the simulated trait has the desired heritability, on average (Fig. 3c). Under a model where the effect sizes are uncorrelated between ancestries, the trans-ancestry meta-analysis approach leads to a slightly reduced power compared to an analysis based only on samples from EUR ancestry, whereas comparable power is estimated for the model of correlated effect sizes (Table 3). These results are driven by the fact that the majority of samples in our study were derived from the EUR ancestry and are not necessarily the case for more balanced sample sizes. The loss of power in the trans-ancestry model in our case arises due to the estimation of three additional parameters (one per additional ancestry) [77] that due to relatively small sample sizes of the non-EUR ancestries are estimated with higher variability.

**Table 3:** Simulation-based power calculations.

*N*	Correlated effect sizes		Uncorrelated effect sizes	
	EUR only	Trans-ancestry	EUR only	Trans-ancestry
100	100% (9)	100% (10)	100% (9.5)	100% (9)
200	100% (7)	100% (7)	100% (7)	100% (6)
300	100% (5)	100% (5)	100% (5)	99% (3)
400	96% (3)	99% (3)	96% (3)	91% (2)
500	94% (2)	91% (2)	91% (2)	74% (1)
750	69% (1)	67% (1)	66% (1)	43% (0)
1000	42% (0)	41% (0)	46% (0)	24% (0)
2000	14% (0)	11% (0)	13% (0)	9% (0)

Power to detect at least one association and the median number of discoveries at genome-wide significance *p* < 5 × 10^−8^, as a function of the number (N) of causal variants (CVs). Each estimate is based on 200 simulated replicates. Between ancestries, the effect sizes of the CVs were either correlated or uncorrelated. The simulated trait has LD-score heritability of 8%.

Our results indicate that if the total number of common CVs explaining an LD score regression-derived $h_{SNP}^2$ of 8.2% was ~500 or less, then the power of our sample to detect at least one association at genome-wide significance level was excellent and generally above 80%, irrespective of the model or the method (Table 3). As a result, our failure to detect any association indicates that the number of CVs explaining 8.2% of the variance is likely to be more than ~750–1000. When heritability is fixed, as the number of CVs increases, the proportion of trait variance explained by each variant decreases, resulting in decreasing power to detect any association. In our case, the power to detect an association with a particular SNV at genome-wide significance was adequate ( > 80%) as long as that SNV explained approximately >0.35% of the trait variance, which can be achieved for various combinations of MAF and effect sizes (Fig. 3d). The fact that we did not detect any association, therefore, indicates that if a common causal SNVs was catalogued by the 1000 Genomes project, or unmeasured but in high LD with one, then this causal SNV is unlikely to explain more than ~0.3% of a trait variance.

## Discussion

Most quantifiable cognitive traits are termed complex due to the fact that they do not follow Mendelian inheritance patterns; instead, they are influenced by a large number of genetic factors including multiple risk alleles, each of a small effect size [87, 88]. Understanding the genetics of inhibitory control is critical for uncovering the genetic architecture of psychiatric and neurodevelopmental disorders such as ADHD that are characterised by significant impairments in a range of executive functions and inhibitory control in particular. Here we performed the first trans-ancestry GWAS using task-based measures of inhibitory control to investigate its genetic architecture. Although we did not identify any genome-wide significant variants, interindividual differences in measures of response inhibition (SSRT) and top-down regulation of attention (GoRT SD) were influenced by genetic factors. Critically, power analyses demonstrated that the lack of significant GWAS associations is due to the number of common causal variants contributing to the heritability of these phenotypes being relatively high and thus larger sample sizes are necessary to robustly identify associations. Linking inhibitory control to the genetics of ADHD we also identified a significant association between ADHD PGRS and reaction time variability, supporting its utility as an endophenotype for ADHD.

Considerable evidence from twin studies indicates moderate heritability for a range of inhibitory control measures [28–34], suggesting that in some tasks more than half of the variance in individual task performance can be explained by genetic factors. These relatively high values are in contrast to more modest heritability estimates accounting for the additive influence of common genetic variation in EFs based on GWASs that commonly do not exceed 30% [51–54]. The discrepancy between twin and DNA-based measures is likely to be related to the effects of rarer genetic variants that are not assessed in GWAS, together with the nonadditive genetic effects [89], whereas another hypothesis suggests that the current estimates of twin-based heritability might be significantly inflated by genetic interactions [90]. Here, for the first time, we estimated a significant SNP-heritability for the measures of inhibitory control (GoRT SD and SSRT, $h_{SNP}^2$ ~8%), exceeding previous evaluations in a smaller sample of 4611 adolescents that failed to find common genetic contributions to stop signal task-based measures [52]. Our study similarly contained a large proportion of children and adolescents (~90%), and thus the overall sample composition with regards to age could also impact heritability estimates as other cognitive domains tend to demonstrate the increased influence of genetic factors later in life compared to childhood [91, 92]. Based on our simulations, we interpret the estimate of $h_{SNP}^2$ = 8.2% as the proportion of variance explained by common (>1%) SNVs catalogued by the 1000 Genomes project (or in high LD with these SNVs). Had we used a denser SNV imputation panel, the SNP heritability might have been higher [93]. At the time the present project was initiated, the only available ancestry-diverse reference panel was from the 1000 Genomes project, however, the use of the larger ancestrally-diverse TOPMed reference panel [94] is encouraged for future research. Overall, our estimates were in line with the prior evidence of heritability of executive function ($h_{SNP}^2$ ~10% in largest samples) [51–54] indicating that the extent of common genetic influences on inhibitory control are comparable to more general factors of EF.

Measures of executive function and inhibitory control in particular have been proposed as endophenotypes for ADHD and, to a lesser extent, schizophrenia and ASD [23–27]. Our findings indicating the significant heritability and identifying the association between ADHD PGS and reaction time variability as well as schizophrenia PGS and reaction time in a large sample of Europeans through meta-analysis further support this idea. We also identified for the first time that PGS for schizophrenia in East Asians (the only summary stats available for this ancestral group) was associated with SSRT. Although ADHD, schizophrenia, and ASD all show deficits in inhibitory control, the polygenic risk for each disorder was differentially associated (or not associated) with the various measures from the stop-signal task in a general population sample. This implies that genetic risk for NDDs may differentially contribute to risk for aspects of executive function and this may vary by ancestral group, although more research is needed to confirm this finding.

The initial search for endophenotypes was based on the assumption that these quantifiable traits should have less complex genetic architectures that are more closely related to gene function [51–54], however, here we demonstrate the inherent complexity of genetic factors contributing to inhibitory control. Through power analyses, we investigated the potential reasons why no genome-wide significant associations were identified, despite observing significant heritability of ~8%. Our findings suggest that the number of common genetic variants explaining the identified heritability is likely to be relatively large exceeding 750–1000, each contributing not more than ~0.3% of the variance. These estimates further support the contention that complex genetic architectures underlie behavioural measures of response inhibition and top-down regulation of attention represented by SSRT and GoRT SD, respectively.

Currently, the protocols for large-scale studies containing genomic data, such as UK Biobank, do not include measures of inhibitory control mainly due to the time required for data collection. In order to achieve adequate sample sizes for a GWAS, data need to be aggregated across multiple studies. Challenges arise due to differences in experimental paradigms of the stop signal task with varying numbers of trials, individual trial lengths, mode of the stop stimuli (visual *vs* auditory), approaches for defining stop signal delay, as well as the methods used for measure estimation. Although it is not possible to retrospectively modify the individual study designs, here we aimed to control the variability in measure estimation by adhering to the best practice protocol proposed by Verbruggen et al., (2019), including the exclusion of subjects that violate the assumptions of the race model, maintaining stop accuracy between 25%-75%, and use of the integration method for SSRT calculation where possible. To minimise variation in the genomic data all study sites used the same reference panel for imputation and imputation quality filter (r^2^ > 0.8). Nevertheless, some variation across study sites remained.

Historically most genomic research focused on genetically homogeneous cohorts from European ancestry populations limiting the generalisability of the identified findings and in some cases leading to biased inferences [95, 96]. Genomic data across different ancestral groups is valuable and increasingly available and will serve to increase the total sample sizes and representativeness of genetic studies. Integrating these data does pose some technical challenges as not all SNPs are polymorphic across different populations, some disease-associated SNPs have vastly different allele frequencies or show marked variability in linkage disequilibrium patterns with the causal variant between populations [97, 98]. Moreover, causal variants might interact with environmental risk factors that differ between ancestral populations additionally generating heterogeneity in the estimated effects. As a result, adjusting for population stratification opposes the goal of maximising the study power as traditional fixed and random effects approaches tend to underestimate the effect sizes or overestimate the standard errors reducing the overall confidence in the identified associations [99, 100]. The lack of non-European GWAS of complex traits, including in psychiatry, limits the ability to conduct polygenic risk score analyses beyond European target samples [101]. Here we demonstrate the first attempt to incorporate data across different ancestries in the meta-analysis of inhibitory control using a method that derives the axes of genetic variation between populations based on genome-wide metrics of diversity via multi-dimensional scaling resulting in increased power over standard approaches while maintaining false positive error rates [77]. Novel approaches for incorporating data from different ancestries are being continuously developed [77, 102–104] providing opportunities for future large-scale trans-ancestry studies to uncover the genetic architecture of complex traits in a generalisable way. The continued inclusion of diverse ancestry, increased recruitment of diverse samples, and GWAS in diverse samples for discovery and polygenic risk score analyses are much needed.

In summary, in this first trans-ancestry GWAS of inhibitory control, we demonstrated that task-derived measures of response inhibition and top-down regulation of attention are influenced by common genetic factors. Importantly, the number of contributing common genetic variants is likely to be relatively large suggesting that larger studies will be required to identify robust genome-wide associations.

### Supplementary information

Supplementary material

Supplementary table 2

Supplementary table 4

## Data Availability

GWAS summary statistics for the trans-ancestry meta-analysis and EUR ancestry meta-analysis are provided at https://tinyurl.com/3w67mfyh.

## Code availability

Genotyping data processing code for SPIT1, SPIT2 and ABCD Study data is provided at http://bitbucket.org/mathieu-lemire/sk-scripts-qc-genotypingarrays. The custom MR-MEGA code implementation is provided at https://bitbucket.org/mathieu-lemire/sk_my_mrmega. Genotype simulation code is provided at https://bitbucket.org/mathieu-lemire/sk_recomb/src/master/.
