# Supplementary material for: Trans-ancestry meta-analysis of genome wide association studies of inhibitory control
Source: Mol Psychiatry. 2023 Jul 27;28(10):4175–84. doi: 10.1038/s41380-023-02187-9 (PMC10827666; doi:10.1038/s41380-023-02187-9)
Supplement: Supplementary file 2 — Supplementary table 2 [file 41380_2023_2187_MOESM2_ESM.pdf]

Chromosome	SNP	Position (hg19)	Non-effect Allele	Effect Allele	N	P-value	Nearest gene(s)	Direction of effect in individual studies														
								AFR	EAS			EUR								SAS		
								ABCD	ABCD	Spit1	Spit2	ABCD	Colorado	IMAGEN	Melbourne	Spit1	Spit2	Michigan	Oregon	ABCD	Spit1	Spit2
GoRT SD																						
4	rs144152868	180,371,794	TA	T	12859	2.79E-07	AC020551.1	+	-	-	?	-	?	-	-	-	?	?	?	-	-	?
4	rs55641303	125,757,777	A	G	7782	3.91E-07	ANKRD50	?	?	?	?	?	-	-	-	-	?	?	?	?	+	?
12	rs150221025	110,180,397	A	ACT	12164	5.20E-07	FAM222A, FAM222A-AS1	+	-	?	?	+	?	-	+	+	?	?	?	?	+	+
9	rs10993885	136,493,802	G	A	13383	6.03E-07	AC002101.1	-	-	+	?	-	-	-	-	-	?	?	?	-	-	?
12	rs66716788	30,887,816	ACACACT	A	14064	6.14E-07	CAPRIN2	+	+	+	-	+	?	+	+	+	+	?	?	+	+	+
16	rs276607	13,174,005	A	C	14320	7.06E-07	SHISA9	+	+	-	-	+	?	+	-	+	+	+	+	-	-	-
10	rs10821860	62,688,004	G	C	11658	7.68E-07	RHOBTB1	?	?	?	?	+	+	-	-	-	?	?	?	-	-	?
6	rs79797664	151,908,423	A	G	13139	8.49E-07	CCDC170	-	+	+	?	-	-	?	-	-	-	?	?	?	-	-
GoRT mean																						
2	rs4140883	59,079,538	C	T	13678	8.90E-08	LINC01122	?	?	+	+	+	-	+	-	+	+	?	?	?	+	-
3	rs142340309	175,925,042	T	TATATAAA	13572	1.64E-07	AC117434.1	?	-	+	+	+	?	+	-	-	-	+	+	+	-	+
10	rs11250961	2,001,914	C	T	14096	2.42E-07	AL355597.1	?	+	+	-	-	+	-	-	-	-	+	-	+	-	-
21	rs2835203	37,351,180	G	A	13639	6.08E-07	RUNX1	-	?	?	?	+	-	-	-	-	+	+	+	+	-	+
SSRT																						
10	rs57532892	104,471,091	GTGTA	G	12158	2.36E-07	ARL3	+	?	?	+	-	?	+	+	-	+	?	?	+	+	?
10	rs60013792	43,389,418	C	T	13272	4.57E-07	LINC02623	?	?	-	+	+	-	-	+	+	+	?	+	+	+	-
12	rs34461970	74,802,358	AT	A	13586	4.72E-07	AC090015.1	-	+	+	+	+	+	+	+	+	+	+	+	+	+	?
8	rs138852029	95,022,417	CAT	C	11918	7.62E-07	AC105081.1	-	?	?	-	-	?	+	?	-	-	+	+	-	?	?
10	rs61831918	1,543,940	A	G	14083	9.45E-07	ADARB2	-	+	+	+	-	+	-	-	-	-	+	+	?	-	-

Supplementary Table 2. Trans-ancestry GWAS suggestive hits. Description of lead SNPs in regions with p<10-6 in the trans-ancestry GWAS meta-analysis, and direction of effect in each individual study. AFR = African ancestry, EAS = East Asian ancestry, EUR = European ancestry, SAS = South Asian ancestry. ABCD = Adolescent Brain Cognitive Development study. For the direction of effect, + indicates a positive relationship between the trait and the number of copies of the effect allele, - indicates a negative relationship, and ? indicates the SNP was not analyzed.
