# Supplementary material for: Trans-ancestry meta-analysis of genome wide association studies of inhibitory control
Source: Mol Psychiatry. 2023 Jul 27;28(10):4175–84. doi: 10.1038/s41380-023-02187-9 (PMC10827666; doi:10.1038/s41380-023-02187-9)
Supplement: Supplementary file 3 — Supplementary table 4 [file 41380_2023_2187_MOESM3_ESM.pdf]

		P-value threshold	0.001			0.05			0.1			0.2			0.3			0.4			0.5		
**GWAS**	**Ancestry**	**Trait**	**Estimate (SE)**	**P-value**	**P-het**	**Estimate (SE)**	**P-value**	**P-het**	**Estimate (SE)**	**P-value**	**P-het**	**Estimate (SE)**	**P-value**	**P-het**	**Estimate (SE)**	**P-value**	**P-het**	**Estimate (SE)**	**P-value**	**P-het**	**Estimate (SE)**	**P-value**	**P-het**
**ADHD**	**EUR**	**GoRT SD**	0.0036 (0.0021)	0.090	0.50	0.0052 (0.0021)	0.013	0.011	0.0063 (0.0021)	0.0029	0.031	0.0068 (0.0021)	**0.0012**	0.027	0.0073 (0.0021)	**0.00053**	0.060	0.0075 (0.0021)	**0.00035**	0.0045	0.0079 (0.0021)	**0.00012**	0.051
		**GoRT mean**	0.00050 (0.0015)	0.74	0.32	0.00032 (0.0015)	0.84	0.076	0.0011 (0.0015)	0.46	0.15	0.0014 (0.0015)	0.38	0.088	0.0018 (0.0015)	0.23	0.10	0.0021 (0.0015)	0.17	0.088	0.0019 (0.0015)	0.21	0.081
		**SSRT**	0.0025 (0.0023)	0.28	0.29	0.00058 (0.0023)	0.80	0.92	0.0013 (0.0023)	0.57	0.90	0.0015 (0.0023)	0.51	0.78	0.0011 (0.0023)	0.63	0.86	0.00078 (0.0023)	0.74	0.87	0.0013 (0.0023)	0.58	0.73
**ASD**	**EUR**	**GoRT SD**	-0.0043 (0.0021)	0.042	0.29	-0.0013 (0.0021)	0.53	0.067	-0.00068 (0.0021)	0.75	0.11	0.00026 (0.0021)	0.90	0.089	0.00049 (0.0021)	0.82	0.15	0.00086 (0.0021)	0.68	0.14	0.00042 (0.0021)	0.84	0.20
		**GoRT mean**	-0.0035 (0.0015)	0.022	0.066	-0.0015 (0.0015)	0.33	0.41	-0.0015 (0.0015)	0.34	0.61	-0.00049 (0.0016)	0.75	0.38	0.000036 (0.0016)	0.98	0.40	0.00041 (0.0016)	0.79	0.27	0.00042 (0.0016)	0.79	0.31
		**SSRT**	-0.0051 (0.0023)	0.027	0.34	-0.00094 (0.0023)	0.69	0.26	-0.0020 (0.0023)	0.39	0.56	-0.0021 (0.0023)	0.38	0.49	-0.0032 (0.0023)	0.17	0.34	-0.0031 (0.0023)	0.19	0.49	-0.0029 (0.0023)	0.22	0.51
**SCZ**	**EUR**	**GoRT SD**	0.0020 (0.0021)	0.35	0.089	0.0018 (0.0021)	0.38	0.029	0.0012 (0.0021)	0.56	0.029	0.0021 (0.0021)	0.31	0.020	0.0020 (0.0021)	0.33	0.053	0.0019 (0.0021)	0.36	0.032	0.0020 (0.0021)	0.34	0.023
		**GoRT mean**	0.0060 (0.0015)	**9.28E-05**	0.29	0.0071 (0.0015)	**3.74E-06**	0.065	0.0072 (0.0015)	**3.39E-06**	0.079	0.0071 (0.0015)	**5.27E-06**	0.11	0.0067 (0.0015)	**1.34E-05**	0.12	0.0066 (0.0016)	**2.25E-05**	0.099	0.0068 (0.0016)	**1.07E-05**	0.071
		**SSRT**	-0.00016 (0.0023)	0.94	0.86	-0.000042 (0.0023)	0.99	0.86	-0.00024 (0.0023)	0.92	0.96	0.00041 (0.0024)	0.86	0.95	-0.0000084 (0.0024)	1.00	0.94	0.000065 (0.0024)	0.98	0.87	0.000074 (0.0024)	0.97	0.88
**SCZ**	**EAS**	**GoRT SD**	0.019 (0.0089)	0.034	0.60	0.019 (0.0088)	0.030	0.37	0.018 (0.0089)	0.048	0.33	0.016 (0.0089)	0.076	0.57667	0.014 (0.0089)	0.13	0.63	0.015 (0.0089)	0.10	0.59	0.014 (0.0089)	0.11	0.60
		**GoRT mean**	0.0096 (0.0053)	0.069	0.33	0.0064 (0.0053)	0.23	0.45	0.0046 (0.0053)	0.39	0.40	0.0017 (0.0053)	0.76	0.59468	0.00091 (0.0053)	0.87	0.64	0.0019 (0.0053)	0.72	0.58	0.0017 (0.0053)	0.75	0.59
		**SSRT**	0.029 (0.011)	**0.0046**	0.22	0.010 (0.010)	0.31	0.80	0.016 (0.010)	0.13	0.70	0.016 (0.010)	0.13	0.61616	0.015 (0.010)	0.13	0.39	0.016 (0.010)	0.13	0.41	0.016 (0.010)	0.13	0.41

Supplementary Table 4. Polygenic score analysis results. Polygenic scores (PGS) were constructed from public GWAS for attention-deficit hyperactivity disorder (ADHD), autism spectrum disorder (ASD) and schizophrenia (SCZ) from sets of clumped SNPs reaching different p-value thresholds. PGS were tested for association with each of 3 traits in each ancestry cohort separately and combined in a meta-analysis. Shown is the effect size (Estimate), standard error (SE), p-value testing if the effect size is zero (P-value), and a p-value testing for heterogeneity across cohorts (P-het). EUR = European, EAS = East Asian. Bold p-values indicate passing a significance threshold adjusted for multiple testing (0.0019 for EUR and 0.0056 for EAS).
